# Effect of Dual Treatment with SDF-1 and BMP-2 on Ectopic and Orthotopic Bone Formation

**DOI:** 10.1371/journal.pone.0120051

**Published:** 2015-03-17

**Authors:** Chang-Hwan Lee, Myoung Uk Jin, Hong-Moon Jung, Jung-Tae Lee, Tae-Geon Kwon

**Affiliations:** 1 Department of Oral & Maxillofacial Surgery, School of Dentistry, Kyungpook National University, Daegu, Republic of Korea; 2 Department of Conservative Dentistry, School of Dentistry, Kyungpook National University, Daegu, Republic of Korea; Kyungpook National University School of Medicine, KOREA, REPUBLIC OF

## Abstract

**Purposes:**

The potent stem cell homing factor stromal cell-derived factor-1 (SDF-1) actively recruits mesenchymal stem cells from circulation and from local bone marrow. It is well established that bone morphogenetic protein-2 (BMP-2) induces ectopic and orthotopic bone formation. However, the exact synergistic effects of BMP-2 and SDF-1 in ectopic and orthotopic bone regeneration models have not been fully investigated. The purpose of this study was to evaluate the potential effects of simultaneous SDF-1 and BMP-2 treatment on bone formation.

**Materials and Methods:**

Various doses of SDF-1 were loaded onto collagen sponges with or without BMP-2.These sponges were implanted into subcutaneous pockets and critical-size calvarial defects in C57BL/6 mice. The specimens were harvested 4 weeks post-surgery and the degree of bone formation in specimens was evaluated by histomorphometric and radiographic density analyses. Osteogenic potential and migration capacity of mesenchymal cells and capillary tube formation of endothelial cells following dual treatment with SDF-1 and BMP-2 were evaluated with in vitro assays.

**Results:**

SDF-1-only-treated implants did not yield significant *in vivo* bone formation and SDF-1 treatment did not enhance BMP-2-induced ectopic and orthotopic bone regeneration. *In vitro* experiments showed that concomitant use of BMP-2 and SDF-1 had no additive effect on osteoblastic differentiation, cell migration or angiogenesis compared to BMP-2 or SDF-1 treatment alone.

**Conclusions:**

These findings imply that sequence-controlled application of SDF-1 and BMP-2 must be further investigated for the enhancement of robust osteogenesis in bone defects.

## Introduction

Autologous bone grafts in bone defect areas are regarded as a gold-standard therapy in clinical practice. Because of donor site morbidity caused by the bone harvesting process, implantation of mesenchymal stem cells (MSCs) from bone marrow into bone defect areas has been suggested as an alternative to bone grafts [1]. MSCs can differentiate into various osteoprogenitor cells that can directly form bone structure and indirectly influence bone regeneration by modulating various growth factors and cytokines; however, the direct use of MSCs for implantation has several limitations. Pluripotent MSCs make up only a small fraction of the host marrow [2] and increasing cell numbers by *ex vivo* cell culture can change the cell characteristics and increase the risk of contamination [3]. The application of cytokines or growth factors at the site of bone healing and regeneration has been suggested as a means of overcoming the shortcomings of cell therapy [4].

In the field of bone tissue engineering, bone morphogenetic protein-2 (BMP-2) is a growth factor known to enhance osteogenesis. BMP-2 actively promotes osteoblastic differentiation and has been approved by the FDA for clinical use in the orthopedic and dental fields [5, 6]. To achieve clinically significant bone regeneration, however, large amounts of BMP-2 are needed [7], while it has been reported that bone formation does not increase dose-dependently with increasing BMP-2 concentrations. In fact, structurally abnormal bone and tissue inflammatory reactions were observed after applications of high concentrations of BMP-2 [8, 9].

Based on the above mentioned effects of BMP-2 on bone regeneration, the application of multiple chemokines or growth factors together with BMP-2 has been suggested in animal model. Growth factors such as vascular endothelial growth factor (VEGF) [10,11] and fibroblast growth factor-2 (FGF-2) [12, 13] have been used concomitantly with BMP-2 to yield higher levels of bone regeneration compared with the use of BMP-2 alone. Recently, the role of stromal cell-derived factor-1 (SDF-1), a chemokine known to mobilize mesenchymal cells to injured tissue, in BMP-2-mediated osteoblastic differentiation was highlighted [14]. SDF-1 plays an important role in the regeneration of various tissues, including bone, and can direct stem cell homing to the site of skeletal injury [15–17]. It is thus logical to postulate that the dual application of the chemokine SDF-1 and the growth factor BMP-2 may activate bone regeneration by activating the mobilization of bone marrow MSCs or circulating MSCs as well as by the potentiation of osteoblastic differentiation. The effect of dual treatment with SDF-1 (0.5μg) and BMP-2 (2.5μg) on collagen disc implantation was previously investigated, and it was found that, compared to treatment with BMP-2 only, dual treatment yielded significantly higher levels of bone formation 4 weeks after subcutaneous scaffold implantation. A dose-dependent increase in BMP-2-induced ectopic bone formation with the application of 0, 0.5, 1, and 5μg SDF-1 was observed [18]; however, subcutaneous implantation of collagen sponge soaked in a combination of SDF-1 (0.2μg) and BMP-2 (10μg) resulted in significantly reduced bone volume compared to collagen sponge soaked in only BMP-2 4 weeks after implantation [19]. In another study, bone regeneration of femoral defects was analyzed after the application of adenovirally activated fat tissue grafts expressing BMP-2 and SDF-1 and cardiac injection of CXCR4-expressing MSCs. Local administration of both BMP-2 and SDF-1 was found to activate more MSC recruitment and bone formation; however, the negative control group exhibited more bone regeneration compared to all experimental groups in this study [20]. Even though several studies have been conducted to investigate the effects of SDF-1 and BMP-2 on osteogenesis in animals, the results of these studies, even when performed under similar conditions, have been somewhat contradictory. Moreover, results from experiments on the ectopic bone formation model alone cannot be directly interpreted as true skeletal repair. It is clear that SDF-1 can actively recruit MSCs and BMP-2 can enhance osteogenesis; however, the optimal conditions for dual application of BMP-2 and SDF-1 have not been clearly demonstrated. There have been very few reports on BMP-2-induced bone regeneration following treatment with various amounts of SDF-1 to date.

The purpose of this study was to investigate the effect of concomitant SDF-1 and BMP-2 treatment on orthotopic and ectopic bone formation. We hypothesized that the combination of SDF-1 and BMP-2 may increase osteogenesis by the cooperative action of these two factors in an *in vivo* animal model, and thus the effects of different doses of SDF-1 on BMP-2-induced *in vivo* bone formation and on *in vitro* osteogenic activity and cell migration were evaluated. The degree of bone regeneration was assessed by histological and radiological analyses in this study.

## Materials and Methods

### Conditions for Animal experiment

Female C57BL/6 mice (7 weeks old) were used to evaluate bone formation in the presence of SDF-1 and/or BMP-2. This experiment was approved from the Institutional Animal Care and Use Committee of Kyungpook National University and all aspects of the animal experiments were conducted in accordance with the approved protocol (2010–30).

The animals were randomly assigned and housed in separate plastic cages and allowed to adapt to the conditions of the animal house for 7 days before the surgery. The number of experimental animal were reached by sample size calculations [21] (by using α = 0.05, power = 0.80 and variability obtained from pilot study). The animals were maintained at about 22 ± 5°C on a 12 h dark/light cycle and allowed free access to tap water and standard laboratory diet and during the experiments. All surgeries were performed on animals under general anesthesia (ketamine: 100 mg/kg body weight; xylazine: 5mg/kg body weight). At the end of the experiments, the animals were euthanized with CO_2_ and the specimens were harvested for further test.

### Reagents and scaffold

Recombinant murine SDF-1α (CXCL12) and human BMP-2 proteins were purchased from PeproTech (Rocky Hill, NJ, USA). SDF-1 (0.1, 0.5, or 1μg) and BMP-2 (2.5μg) inphosphate-buffered saline (PBS; final volume: 50μl) were loaded onto resorbable atelo-collagen sponges (Teruplug, Terudermis Olympus Terumo Biomaterials Co.;Tokyo, Japan). Control- (PBS only) and cytokine-loaded scaffolds were used in experiments on orthotopic and ectopic bone formation models.

### Ectopic bone formation following subcutaneous implantation

To evaluate the effects of different doses of SDF-1 on BMP-2-induced ectopic bone formation, cytokine-loaded collagen sponges were implanted subcutaneously in mice. Experimental animals (n = 40) were divided into 8 groups (n = 5 animals per group). Under general anesthesia, an incision was made on the dorsal surface of the animal and subcutaneous pockets were made bilaterally. The same type of scaffold was bilaterally implanted in each animal and thus a total of 80 collagen scaffolds were implanted and were loaded with PBS or BMP-2 and/or SDF-1. The treatment groups were as follows: (1) scaffold only (PBS), (2) 0.1μg SDF-1, (3) 0.5μg SDF-1, (4) 1μg SDF-1, (5) 2.5μg BMP-2, (6) dual treatment with0.1μg SDF-1 and 2.5μg BMP-2, (7) dual treatment with 0.5μg SDF-1 and 2.5μg BMP-2, and (8) dual treatment with 1μg SDF-1and 2.5μg BMP-2. Specimens were retrieved from subcutaneous pockets 28 days post-surgery and fixed in 4% paraformaldehyde at 4°C for subsequent analysis.

### Orthotopic bone formation at critical-size calvarial bone defects

To evaluate the effects of SDF-1 and BMP-2 dual treatment on orthotopic bone formation, an experimental design similar to that used in the ectopic bone formation experiments was applied in a mouse calvarial defect model. Mice (n = 32) were divided into 8 groups (n = 4 per group):(1) scaffold only (PBS), (2) 0.1μg SDF-1, (3) 0.5μg SDF-1, (4) 1μg SDF-1, (5) 2.5μg BMP-2, (6) dual treatment with 0.1μg SDF-1and 2.5μg BMP-2, (7) dual treatment with 0.5μg SDF-1 and 2.5μg BMP-2, and (8) dual treatment with1μg SDF-1 and 2.5μg BMP-2. The PBS- or cytokine-loaded collagen sponges were applied on critical-size calvarial defects (4 mm in diameter) in parietal bone. Calvarial specimens including the defect areas were harvested 28 days post-surgery and processed in the same way as the ectopic bone specimens.

### Radiological and histological evaluations of bone formation

Harvested subcutaneous implants and calvarias including defects were evaluated radiographically and histologically to quantify bone regeneration. X-ray imaging was performed using a soft X-ray unit (Faxitron Specimen Radiography System; Tucson, AZ, USA) at 24 Kvp and 2.5 mA for 20 s. Radiodensity in the regenerated areas was calculated from the captured X-ray images of specimens using Image J software.

After x-ray imaging, fixed specimens were decalcified in EDTA and embedded in paraffin. Sections (10 μm thick) of embedded specimens were stained with trichrome and hematoxylin-eosin (H&E) according to standard protocols. Image analysis of bone formation area was carried out using i-solution software (Image & Microscope Tech.; Daejeon, Republic of Korea). Areas of newly formed bone (mm^2^) and ratios of new bone formation (%, [new bone area/total histological tissue area]) and were analyzed histomorphometrically.

### *In vitro* effects of SDF-1 and BMP-2 on osteogenic differentiation and cell migration

To further evaluate the effects of SDF-1 on osteoblastic differentiation, alkaline phosphatase (ALP) activity was measured in C3H10T1/2 cells (murine pluripotent MSCs; American Type Culture Collection, Manassas, VA, USA). Briefly, C3H10T1/2 cells were seeded (5 × 10^4^ cells/well in triplicate 12-well plates) and incubated in osteogenic media (10% fetal bovine serum with 1%antibiotics in Dulbecco’s modified Eagle medium (DMEM), 10 mM β-glycerol phosphate, and 50g/ml L-ascorbic acid 2-phosphate) for 8 days. Various concentrations of SDF-1 (0, 0.1, 0.5, or 1μg/ml) were included in the media in the presence or absence of BMP-2 (0.5μg/ml). Cells were harvested in ProteoJET lysis buffer (Fermentas; Burlington, Canada). ALP activity was measured according to the manufacturer’s instructions and the resulting absorbance (405 nm) values were normalized to the total protein content in each sample.

The *in vitro* effects of SDF-1 and/or BMP-2 treatment on MSC migration were tested using a transwell migration assay [22]. C3H10T1/2 cells were suspended in 100μl serum-free DMEM (3 × 10^5^cells/well) and seeded into the upper chambers of transwell dishes (Corning Transwell, Sigma-Aldrich; St. Louis, MO, USA), while 400 μl media containing 2% FBS was placed into the lower chambers. After 24 h, the medium in the lower chambers of the transwell dishes was replaced with 400 μl serum-free DMEM with/without 0.5μg/ml SDF-1 and/or 0.5μg/ml BMP-2 (control; SDF-1 only; BMP-2 only; both SDF-1 and BMP-2). After an additional 24 h of incubation (37°C), transwell filters were gently washed with PBS and fixed with 600μl methanol for cell migration assessment. Cell adhered to the filters were stained with 600μl crystal violet (Junsei Chemical Co., Japan) for 30 min at room temperature and cells were quantified by counting cells in five random visual fields at 100× magnification under a light microscope. All *in vitro* experiments were performed in triplicate across three independent experiments.

### HUVEC tube formation assay for *in vitro* angiogenesis

The 96-well plate were coated with cold Matrigel (Corning Martrigel Basement Matrix, Corning incorporated Life sciences, MA, USA) that was diluted by the endothelial growth basal medium (EBM-2, Lonza, MD, USA). After incubation of 1 h at 37°C, HUVECs (2 X 10^4^ cells/well) were seeded to Matrigel-coated wells in EBM-2 with/without 0.5μg/ml SDF-1 and/or 0.5μg/ml BMP-2 (control; SDF-1 only; BMP-2 only; both SDF-1 and BMP-2). Four hours later, three non-overlapping microscopic images in each well were randomly photographed at low-power magnification (x 4). The observed total tube length and branching points formed by endothelial cells per image field were measured by using i-solution software (Image & Microscope Tech.; Daejeon, Republic of Korea).

### Statistical analysis

All data were statistically evaluated by analysis of variance (ANOVA) followed by Tukey’s post-hoc test to evaluate differences between treatment groups using SPSS PC 10 software (SPSS Inc.; Chicago, IL, USA). The level of significance was set at *p*< 0.05.

## Results

### The effects of SDF-1 and BMP-2 treatment on ectopic bone formation

**Fig 1:**
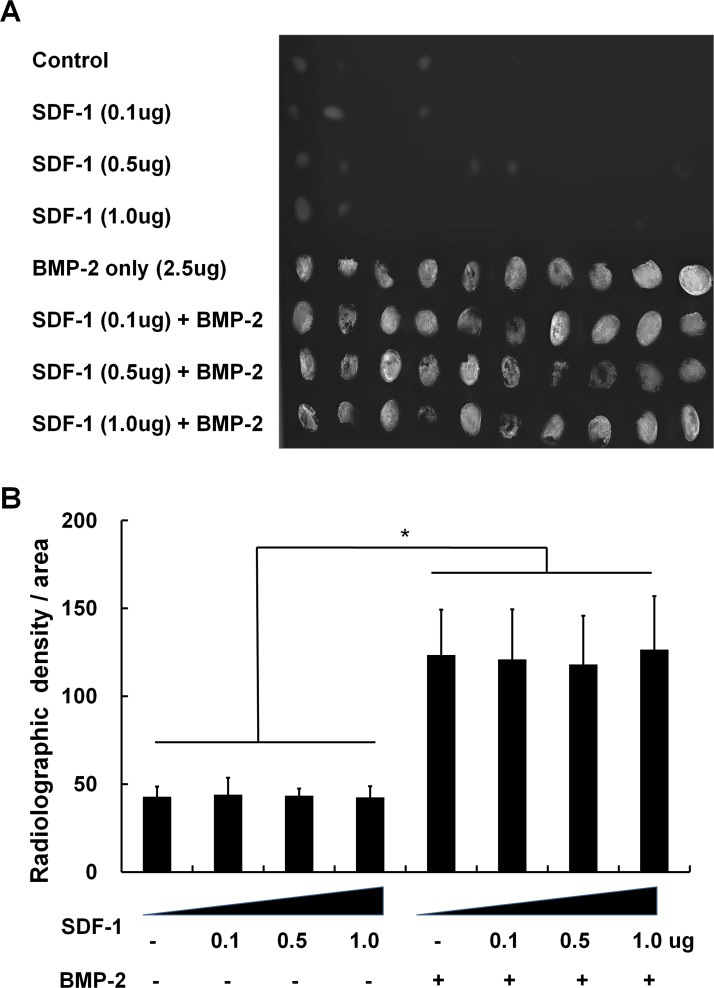
The effect of simultaneous SDF-1 and BMP-2 treatment on ectopic bone formation. Faxitron soft X-ray images after subcutaneous implantation of collagen sponge with/without BMP-2 (2.5μg) and SDF-1 (0, 0.1, 0.5, or 1 μg/collagen sponge) in mice. (A) Radiographic image of the harvested collagen sponges 4 weeks after implantation. (B) Comparison of radiological density of collagen sponges from different treatment groups (*, *p* < 0.05).

All the animals maintained healthy condition and did not experienced adverse events after the surgery. Soft X-ray evaluation after subcutaneous collagen implantation into mice revealed that SDF-1 (0.1, 0.5, and 1 μg) application did not significantly enhance bone formation by 4 weeks after implantation. SDF-1-treated samples were found to have similar radiographic densities compared with control samples (empty collagen sponges). BMP-2, on the other hand, greatly enhanced ectopic bone formation. This BMP-2-induced ectopic bone formation was not enhanced by SDF-1 treatment at any of the doses tested (Fig. 1).

**Fig 2:**
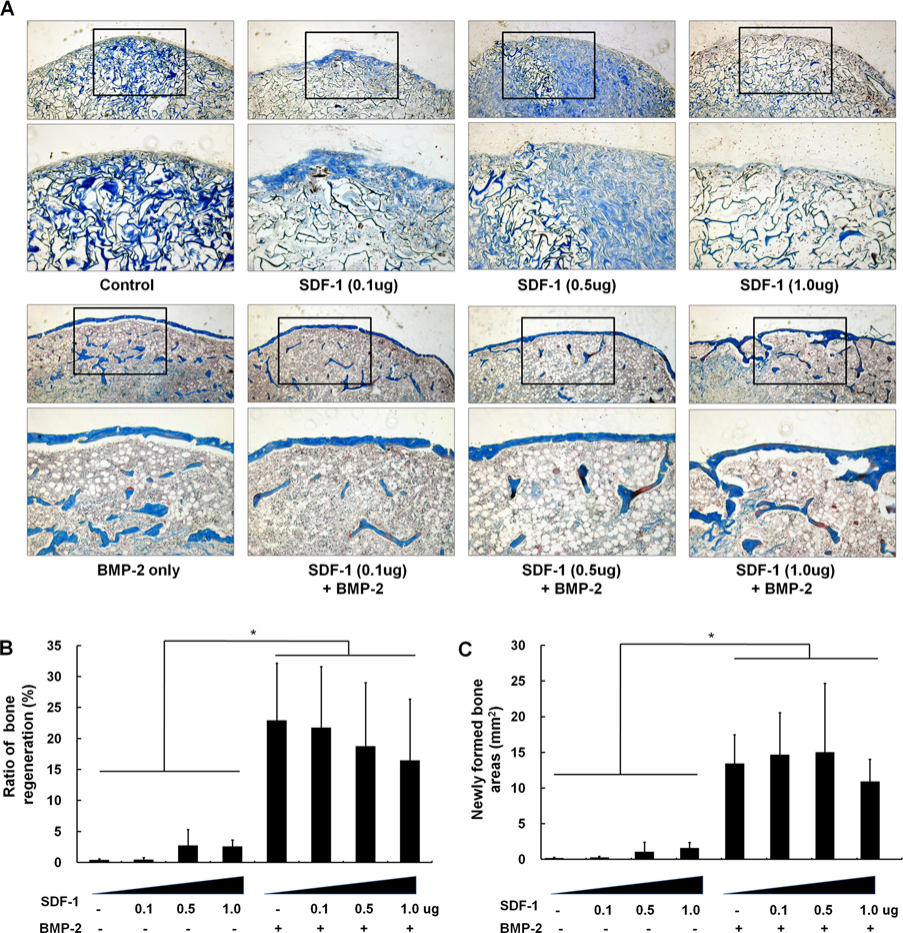
Histological findings of ectopic bone formation after treatment with various concentrations of SDF-1 with/without BMP-2. (A) Representative histological sections 4 weeks after surgery (trichrome staining, ×40 in the box). (B) Bone formation ratio (%) and newly formed bone area (mm^2^) were analyzed histomorphometrically. Concomitant BMP-2 and SDF-1 treatment did not significantly activate ectopic bone formation compared to independent treatment with BMP-2 or SDF-1 alone (*, *p* < 0.05).

Similar to the findings of radiographic density assessments, histological examination of subcutaneous implants showed that BMP-2 treatment resulted in significantly more bone formation than control conditions. Treatment with 1μg SDF-1 alone resulted in 2.6% new bone formation, whereas 2.5μg BMP-2 application alone resulted in 22.9 ± 9.2% new bone formation (13.4 ± 4.1mm^2^ newly formed bone). Bone formation (mm^2^) and proportion of newly formed bone (%) was slightly enhanced by higher doses of SDF-1 compared with empty collagen sponges, although these differences were not statistically significant (new bone formation areas: 1.6mm^2^ in 1μg SDF-1-treated samples compared with negligible area in empty scaffold samples). Interestingly, there was tendency that a high dose (1μg) of SDF-1 in conjunction with BMP-2 treatment resulted in reduced bone formation compared to BMP-2 treatment alone. However, because of high standard deviation, the difference did not show statistical significance. These findings indicate that concomitant BMP-2 and SDF-1 treatment did not significantly activate ectopic bone formation compared to BMP-2 treatment alone (Fig. 2).

### The effect of SDF-1 and BMP-2 treatment on orthotopic bone formation

**Fig 3:**
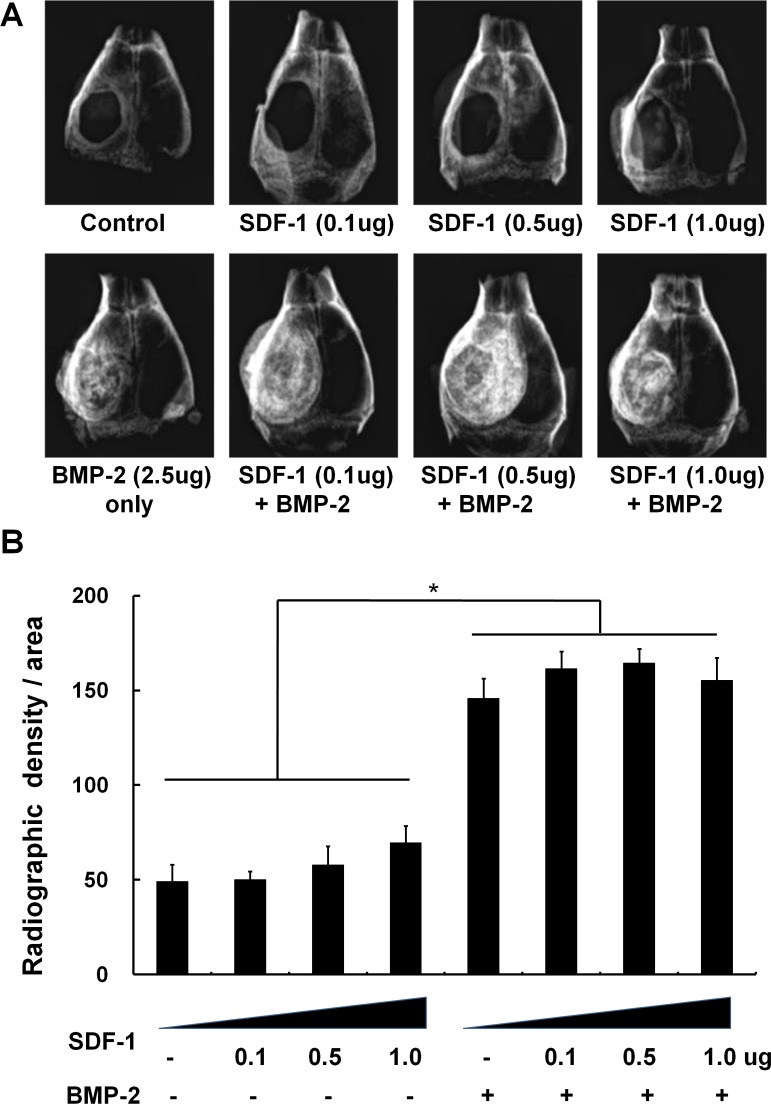
The effect of simultaneous treatment with SDF-1 and BMP-2 on orthotopic bone formation. Soft X-ray examination was carried out after implantation of collagen sponges to critical-size calvarial defects with/without BMP-2 (2.5μg) and SDF-1 (0, 0.1, 0.5, or 1 μg/collagen sponge) in mice. (A) Representative radiographic images, (B) Quantification of bone regeneration by radiographic density at 4 weeks post-implantation (*, *p* < 0.05).

Similar to the ectopic bone formation model, all BMP-2-treated samples exhibited significantly more orthotopic bone formation compared with all samples lacking BMP-2 treatment in radiographic assessment. Treatment with 1μg SDF-1 resulted in slightly increased (31.8%) cranial bone formation at the critical-size calvarial defect compared to 0.1μg SDF-1treatment (*p* < 0.05); however, SDF-1 did not increase BMP-2-induced bone formation at any of the doses tested (Fig. 3).

**Fig 4:**
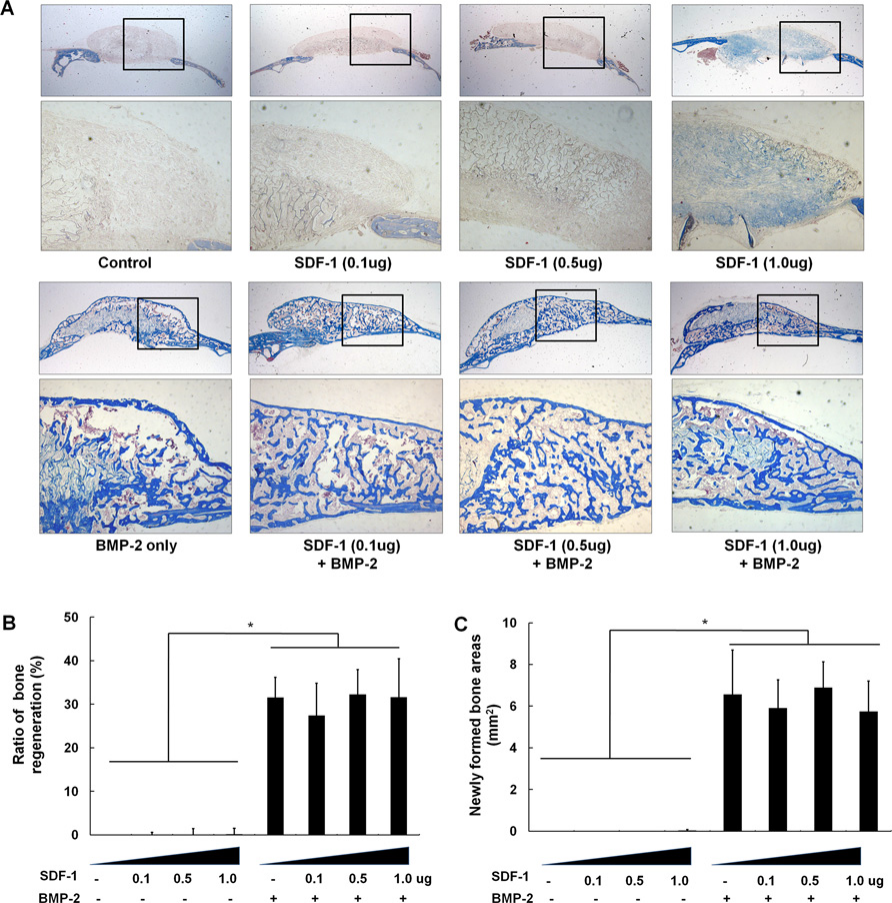
Histological findings of orthotopic bone formation after treatment with various concentrations of SDF-1 with/without BMP2. (A) Representative histological sections 4 weeks post-surgery (trichrome staining, ×40 in the box). (B) Bone formation ratio (%) and newly formed bone area (mm^2^) were analyzed histomorphometrically. Concomitant BMP-2 and SDF-1 treatment did not significantly activate orthotopic bone formation compared to independent treatment with BMP-2 or SDF-1 alone (*, *p* < 0.05).

Histological analysis revealed that treatment with SDF-1 resulted in the formation of statistically insignificant amounts of new bone (0.1, 0.5, and 1μg doses resulted in 0.01 ~0.21mm^2^ of new bone). Consistent with the soft X-ray imaging results, BMP-2 treatment was found to induce significant amounts of new bone formation in terms of new bone area (6.6 ± 2.1mm^2^) and ratio (31.6 ± 4.9%) in cranial bone defects; however, this induction was not enhanced by the addition SDF-1. No statistical differences were observed in bone formation among the BMP-2-treated groups with different doses of SDF-1 (Fig. 4).

### *In vitro* osteogenic differentiation and cell migration after treatment with SDF-1 in the presence or absence of BMP-2

**Fig 5:**
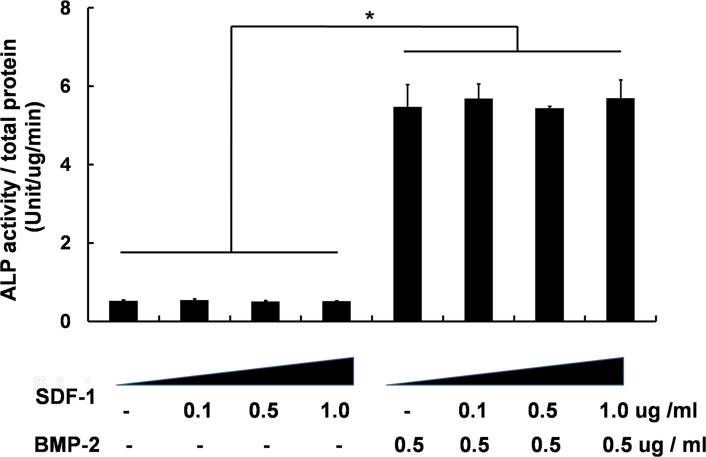
Effect of SDF-1 on ALP activity in C3H10T1/2 mesenchymal cells under osteogenic induction. SDF-1 did not significantly enhance BMP-2-induced osteoblastic differentiation after 10 days of treatment (*, *p* < 0.05).

To further investigate the effects of dual treatment with SDF-1 and BMP-2 on osteogenic differentiation *in vitro*, murine mesenchymal cells (C3H10T1/2) were cultured for 10 days under the same treatment conditions used in the *in vivo* animal experiments. Osteoblastic differentiation was measured by assessing ALP activity after treatment with various doses of SDF-1 in the presence and absence of BMP-2. In accordance with previous reports [23, 24], SDF-1 alone was found not to influence osteoblastic differentiation and in keeping with our *in vivo* data, BMP-2-induced osteogenic activity was not altered by SDF-1 treatment (Fig. 5).

**Fig 6:**
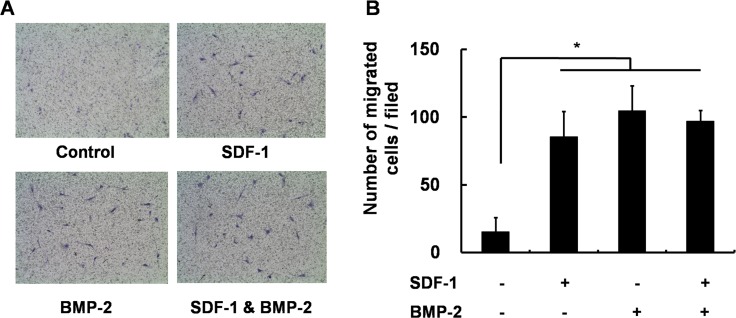
Transwell migration assay. (A) Representative microscopic images of cells cultured in media containing SDF-1 alone, BMP-2 alone, SDF-1 + BMP-2, or neither factor. (B) Quantification of migration of C3H10T1/2 after 24h treatment with the indicated conditions. Treatment with SDF-1 (0.5μg/ml) and BMP-2 (0.5μg/ml) significantly promoted *in vitro* cell mobility (*, *p* < 0.05), while concomitant SDF-1 and BMP-2 application did not further enhance the cell migration.

Next, we investigated the effects of SDF-1 and/or BMP-2 treatment on cell migration in C3H10T1/2 cells. SDF-1 was previously shown to mediate progenitor cell migration [16], while BMP-2 is also known to stimulate chemotactic cell migration [25, 26]. We therefore investigated whether co-treatment of C3H10T1/2 cells with SDF-1 and BMP-2 has a synergistic effect on cell migration. Our findings revealed that treatment with either SDF-1 or BMP-2 does induce migration in these cells; however, concomitant SDF-1 and BMP-2 application did not further elevate *in vitro* cell migration compared with single treatment (Fig. 6).

### Effect of chemokines on angiogenesis *in vitro*

**Fig 7:**
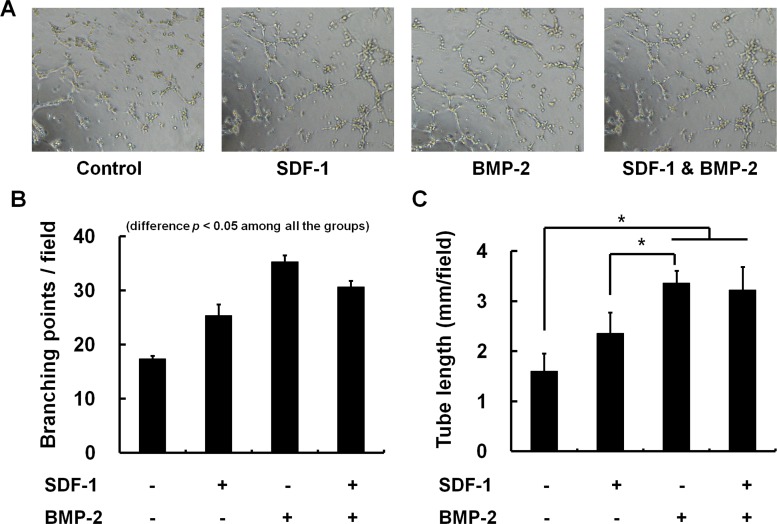
Tube formation assay for angiogenesis on Matrigel. Effect of SDF-1 and BMP-2 on *in vitro* angiogenesis was evaluated with HUVEC tube formation assay. The tube formation after treatment with SDF-1 alone, BMP-2 alone, combined SDF-1 & BMP-2 were microscopically compared (A). The number of branching point per field (B) and total length of tube per field (C) were quantified by counting 3 random fields/well under the microscope (x4). Representative photos of HUVEC tube formation assay reveal that BMP-2 or SDF-1 alone could significantly activate angiogenesis (*, *p* < 0.05). However, co-treatment of BMP-2 and SDF-1 did not show additive effect on antigenic tube formation compared to a single treatment of BMP-2.

Tube formation assays was carried out to investigate the influence of SDF-1 and/or BMP-2 on endothelial cell differentiation into a capillary-like structure, which is a major step for angiogenic process. HUVECs on matrigel formed significantly greater vessel network in the presence of BMP-2 (0.5ug/ml) or SDF-1 (0.5ug/ml) (*p* < 0.05). However, combined SDF-1 or BMP-2 did not show additive effect on tube network formation. The number of branching point per field was even decreased in SDF-1 & BMP-2 co-treated cells compared to the BMP-2-only treated condition (*p* < 0.05) (Fig. 7).

## Discussion

The purpose of this study was to evaluate whether co-treatment with the active stem cell mobilizer SDF-1 and the well-known osteogenic growth factor BMP-2 enhances ectopic and orthotopic bone formation following implantation in a mouse model. We hypothesized that application of concommitent SDF-1 (0.1, 0.5, or 1μg) and BMP-2 (2.5μg)-loaded acellular collagen sponges in critical-size calvarial defects or subcutaneous pockets on the backs of mice would enhance more increased bone formation than control or BMP-2-only treated group.

The role of SDF-1 in stem cell mobilization has previously been reported: the interaction between SDF-1 and its receptor CXCR4 is important in hematopoietic stem cell [27] and bone marrow-derived osteoblast cell mobilization [15]. In a mouse fracture model, SDF-1 has been shown to promote bone regeneration by recruiting MSCs [16] or endothelial precursor cells [28] to the injured bone. The chemotactic effect of SDF-1 in potentiating the migration of host MSCs and enhancing osteochondral defect healing have been demonstrated *in vivo* [29]. *In vitro* MSC migration is furthermore known to increase with increasing doses of SDF-1 [16, 24] and many studies have therefore been conducted to investigate the potential role of SDF-1 in bone regeneration. When retrovirally induced high SDF-1-expressing bone marrow stromal cells were combined with allograft particles and implanted into subcutaneous pockets in mice, remarkable enhancement of ectopic bone formation was observed 8 weeks after surgery. Liu et al. showed treatment with SDF-1-alone to result in slightly increased but statistically insignificant new bone volume in mouse calvarial defects [24]. It is clear that SDF-1 is closely associated with survival and growth of MSCs [30]; however, direct application of SDF-1 does not promote *in vitro* osteogenic differentiation [28,30]. Bone marrow-derived MSCs treated with SDF-1 (0.05, 0.1, 0.2, and 0.4 μg/ml) were not found to have altered ALP activity compared to control cells [24], and similarly, it was shown in this study that SDF-1 (0.1, 0.5, and 1 μg/ml) does not promote *in vitro* osteogenic differentiation. Taken together with previous reports, our findings therefore indicate that the previously known effect of SDF-1 on *in vivo* bone formation is an indirect effect resulting from increased cell mobilization.

In ectopic bone formation models, there are theoretically few pre-existing MSCs present in subcutaneous pockets that readily differentiate into osteoprogenitor cells. The possible stimulatory effect of mechanical force from bone or signaling cascades is furthermore nearly absent in subdermal environment [31]. Nearly 40% of the circulating osteoprogenitor cells in subcutaneous implants are derived from the bone marrow in ectopic bone formation models [32]. Therefore, bone formation in subcutaneous defects is more challenging compared to the orthotopic model of calvarial bone defects, in which bone injuries are surrounded by periosteum and bone marrow and are thus indirect contact with local MSCs. Thus, we used both ectopic and orthotopic bone formation models to assess the effects of SDF-1 treatment on bone regeneration and the influence of SDF-1 on BMP-2-induced bone formation. By using these two animal models, the result of the study would be more readily translated to preclinical condition.

Our investigation into whether or not SDF-1 increases bone formation in the two animal models described above showed that there was nearly no bone formation by 4 weeks after implantation of collagen scaffolds loaded with different doses of SDF-1. This finding is in accordance with previous reports. Ratanavaraporn et al. reported that gelatin hydrogel impregnated with 5μg of SDF-1 or PBS resulted in nearly no bone formation in a rat subcutaneous pocket and ulnar critical-size defect by 4 weeks post-surgery [23]. In a mouse model of critical-size femoral defect, MSCs over-expressing SDF-1 did not yield higher bone formation compared with controls [20]. Recently, Jin et al [33] conducted an investigation into the effects of SDF-1 on ectopic and orthotopic bone formation similar to that carried out in this study and showed that treatment with 1μg SDF-1 does not enhance murine ectopic bone formation, which is in agreement with our findings. SDF-1 treatment in the study by Jin et al. was, however, shown to promote BMP-7-induced bone regeneration in calvarial defects by ~55% mineral content relative to BMP-7-induced calvarial bone formation in the absence of SDF-1 treatment. This discrepancy may be attributed to different growth factors (BMP-2 versus BMP-7) and scaffolds (collagen versus demineralized insoluble bone matrix) used in the two studies.

As an active osteogenic regulator, BMP-2 induces ectopic and orthotopic bone formation and is furthermore known to enhance cell chemotaxis and angiogenesis [34,35]. Considering these known effects of BMP-2, it is thus conceivable that combined SDF-1 and BMP-2 treatment in bone defects may result in more accelerated bone regeneration compared to treatment with either BMP-2 or SDF-1 alone. However, the results of *in vivo* experiments testing this hypothesis have been somewhat contradictory. By 4 weeks after athymic rat subcutaneous implantation, collagen sponges loaded with 0.5μg SDF-1 and 2.5μg BMP-2 promoted bone formation compared with sponges loaded with BMP-2 alone [18]. Findings of the same study also suggested that SDF-1 increases BMP-2-mediated ectopic bone formation in a dose-dependent manner. Under similar experimental conditions, however, co-treatment with 0.2μg SDF-1and 10μg BMP-2 in rat subcutaneous model did not yield elevated new bone volume and bone mineral content compared with single treatment with BMP-2 alone by 4 weeks post-surgery; but after 8 weeks, combined treatment was shown to yield enhanced bone formation compared with single treatment with BMP-2 [19]. Interestingly, BMP-7-induced mouse ectopic bone formation has even been shown to decrease with high doses of SDF-1 (10μg) [33]. The findings of our ectopic bone formation experiments showed that SDF-1 did not increase BMP-2-induced new bone formation. These differences may be attributed to differences between the experimental species, time frame of the experiments, and amount of cytokines of our study and previous studies, which implies that potential synergistic effects of SDF-1 and BMP-2 require very specific experimental conditions.

We also tested whether SDF-1 and BMP-2 exert synergistic effects to promote bone regeneration in calvarial defects and, similar to our findings in the ectopic bone formation experiments, an additive effect was not observed. There are several potential explanations for this observed lack of synergy. First, specific dose condition may be required for these two factors to act synergistically. Our *in vitro* cell migration assay and angiogenic tube formation assay also showed that both SDF-1 and BMP-2 independently increase cell mobility or tube-like structure formation, but that co-treatment with both factors does not further activate cell chemotaxis or angiogenesis. There is possibility that *in vitro* chemotactic or angiogenic activity might be reached to a maximum limit under a single factor (BMP-2) alone. This potential limitation need to be investigated further with more experimental subjects treated with varing dose of the BMP-2 and SDF-1. The second possible explanation for the absence of additive effects observed in this study is that the timing of the SDF-1 and BMP-2 treatments is important for significant changes in osteogenesis to be observed. It is interesting to note that SDF-1 expression is down-regulated in mature osteogenic cell populations and decreases under osteogenic culture conditions [30] and that MSCs from periodontal ligaments exhibit decreased SDF-1 secretion after osteogenic induction [36]. SDF-1 expression is further more absent in osteocytes within the bone matrix [37], and therefore the effect of the sequence in which the two factors are administered for enhanced regeneration needs to be further investigated.

In this study, ectopic and orthotopic bone formation models were used to show that treatment with SDF-1 alone does not enhance bone formation. In addition, SDF-1 treatment does not further enhance BMP-2-induced bone regeneration *in vivo*. *In vitro* assays further more showed that co-treatment of cells with BMP-2 and SDF-1 had no additive effect on osteoblastic differentiation, cell migration or angiogenesis compared to independent treatment with each factor. We conclude from these findings that further experiments investigating various dose- and temporally controlled applications of SDF-1 and BMP-2 are required to demonstrate the expected synergistic effects of the two factors on ectopic or orthotopic bone regeneration.
